# Endoplasmic reticulum autophagy in inflammatory diseases

**DOI:** 10.3389/fimmu.2026.1822159

**Published:** 2026-07-06

**Authors:** Chunxiao Wang, Jing Gao, Changsheng Guo, Qi Li, Feixiao Wang, Ming Zhang

**Affiliations:** 1 Department of Rehabilitation Medicine, Zhengzhou Eighth People's Hospital, Zhengzhou, China; 2 First Affiliated Hospital of Henan University of Traditional Chinese Medicine, Zhengzhou, China; 3 Heilongjiang University of Chinese Medicine, Heilongjiang, China; 4 Henan University of Chinese Medicine, Zhengzhou, China

**Keywords:** endoplasmic reticulum, ER stress, ER-associated degradation, ER-phagy, inflammation, unfolded protein response

## Abstract

The endoplasmic reticulum (ER) is responsible for the synthesis, modification, and folding of various intracellular proteins. Under strong external stimuli, the ER often undergoes significant structural disorganization and functional abnormalities, a process that generally accelerates the onset and progression of inflammatory responses and related diseases, such as infections and sepsis, digestive system diseases, cancer, neurological disorders, circulatory diseases, and musculoskeletal diseases. Therefore, maintaining ER homeostasis is crucial for delaying the inflammatory process. As an important type of selective autophagy, ER-phagy has transcended merely “waste removal” to become a key cellular hub integrating immune and stress signals. It not only effectively curbs the excessive activation of the NF-κB pathway and the NLRP3 inflammasome by timely clearing inflammatory pathogens and ER fragments damaged by calcium store abnormalities and oxidation but also maintains immune cell homeostasis, thereby inhibiting the initiation and spread of excessive inflammatory responses. This review summarizes the key receptors, regulatory mechanisms, and the latest research on ER-phagy in various inflammation-related diseases, aiming to draw academic attention to the important value of ER-phagy in inflammatory diseases.

## 1 Introduction

The endoplasmic reticulum is a vast membrane system composed of flattened cisternae and dynamic tubules, playing key roles in protein quality control, lipid synthesis, Ca^2+^ homeostasis, and inter-organelle communication (1). ER morphology is not static but undergoes continuous dynamic remodeling to adapt to changing cellular environments (2). To maintain its functional homeostasis, cells have evolved a sophisticated ER quality control system, primarily including the unfolded protein response (UPR), ER-associated degradation (ERAD), and ER-phagy (3). When cells encounter stimuli such as nutrient deprivation, oxidative stress, or Ca^2+^ homeostasis imbalance, the protein folding capacity of the ER is impaired, leading to the accumulation of unfolded or misfolded proteins. In response to this crisis, cells rapidly initiate the UPR signaling pathway (4) to sequester potentially toxic unfolded proteins into specific regions, creating conditions for subsequent ERAD processing. When misfolded proteins exceed the degradative capacity of ERAD, the highly selective ER-phagy process is activated, which is a core link in maintaining ER dynamic balance (5). However, when ER damage is too severe or persistent, this protective mechanism may shift to “maladaptive UPR” or ER overload response (6), thereby triggering pro-inflammatory and pro-apoptotic signals.

In recent years, research has increasingly revealed a tight and complex bidirectional interaction between ER-phagy and inflammatory responses. The accumulation of large amounts of unfolded or misfolded proteins due to Ca^2+^ homeostasis imbalance, excessive Reactive Oxygen Species (ROS) production, and impaired ER-phagy cooperatively induces cell apoptosis via the CHOP/caspase-12 pathway, releasing damage-associated molecular patterns (DAMPs) such as HMGB1 and promoting the activation of the NF-κB pathway and the NLRP3 inflammasome, thus triggering inflammatory responses (7–9). However, ER-phagy effectively curbs the excessive activation of the NF-κB pathway and the NLRP3 inflammasome by timely clearing inflammatory pathogens and ER fragments damaged by calcium store abnormalities and oxidation while also maintaining immune cell homeostasis, inhibiting the initiation and spread of excessive inflammatory responses (10–12).

In summary, ER-phagy has surpassed pure “waste removal” to become a key cellular hub integrating immune and stress signals. This review aimed to systematically summarize the key receptors, regulatory signals, and pathological significance of ER-phagy in inflammatory responses and related diseases, providing new ideas and targets for the prevention and treatment of inflammatory diseases.

## 2 ER quality control

ERAD and the UPR are two key intracellular quality control mechanisms. The UPR responds to the accumulation of misfolded proteins and activates ERAD and ER-phagy, while ERAD is responsible for clearing misfolded proteins in the ER and degrading them via the proteasome.

### 2.1 UPR

As the core defense mechanism of cells against ER stress, the UPR enhances ER protein folding capacity by activating the three major branches of ERN1/IRE1, PERK, and ATF6 (13) and timely upregulates ERAD and ER-phagy under severe stress to clear misfolded proteins and restore cellular homeostasis.

In the early stage of stress, PERK, as a transmembrane kinase, is activated and phosphorylates downstream eIF2α, converting it from active to inactive, inhibiting global protein synthesis to reduce ER burden. At the same time, phosphorylated eIF2α selectively promotes the translation of ATF4 mRNA; ATF4 enters the nucleus and initiates the transcription of ER stress-related genes, such as CHOP and GADD34, participating in stress repair or apoptosis regulation (14). ATF6, upon activation, translocates to the Golgi apparatus, where it is cleaved by S1P and S2P proteases to release its active cytoplasmic fragment ATF6(N) (or p50). This fragment enhances XBP1 transcription and, together with XBP1s produced by IRE1 splicing, enters the nucleus to promote the expression of ERAD components such as EDEM and HRD1 and protein folding-related genes, thereby enhancing ER protein folding and degradation capacity to alleviate ER stress (14, 15).

If stress persists, the UPR further initiates ER-phagy to clear damaged components in bulk. Specifically, the PERK–eIF2α–ATF4 signaling axis can induce the expression of ER-phagy receptors, such as CCPG1, SEC62, and TEX264 under stress (16–18), while IRE1 activity is enhanced when PERK is inhibited (16), and its splicing product XBP1s can upregulate the expression of RTN3L and FAM134B (19, 20); stress-activated transcription factor ATF6, after entering the nucleus, cooperates with XBP1 to promote membrane lipid synthesis, providing a necessary structural basis for subsequent ER-phagy (21).

### 2.2 ERAD

ERAD is an important pathway that retrotranslocates misfolded proteins to the cytoplasm and then degrades them via the ubiquitin–proteasome system (22). Based on substrate localization, ERAD can be divided into three pathways: ERAD-L, ERAD-M, and ERAD-C (23). ERAD-L uses the E3 ligase HRD1 complex to degrade misfolded integral membrane or soluble proteins in the ER lumen, a process dependent on membrane proteins HRD3, Usa1D, and DER1 and the luminal protein Yos9; ERAD-M uses the HRD1 complex to degrade misfolded integral membrane proteins in the ER transmembrane domain; ERAD-C specifically degrades misfolded integral membrane proteins on the cytoplasmic side of the ER, but its function requires another E3 ligase, Doa10 (24).

ERAD typically goes through five stages: recognition, targeting, retrotranslocation/extraction, ubiquitination, and degradation (25). That is, ERAD recognizes and targets misfolded proteins in the ER for degradation, retrotranslocates the target proteins from the ER lumen or membrane to the cytoplasm, ubiquitinates them, and finally degrades them by the 26S proteasome (26, 27). EDEM plays a key role in this process by demannosylating misfolded glycoproteins, which are then recognized by the mannose-binding lectins OS-9/XTP3-B and delivered to the ER membrane-embedded E3 ubiquitin ligase complex, ultimately degraded via the Ubiquitin- Proteasome System (UPS) (28). When ERAD is impaired and soluble misfolded proteins cannot be efficiently cleared, cells activate ER-phagy (28), directly targeting ER subdomains containing misfolded proteins to lysosomes for degradation (29).

**Figure 1:**
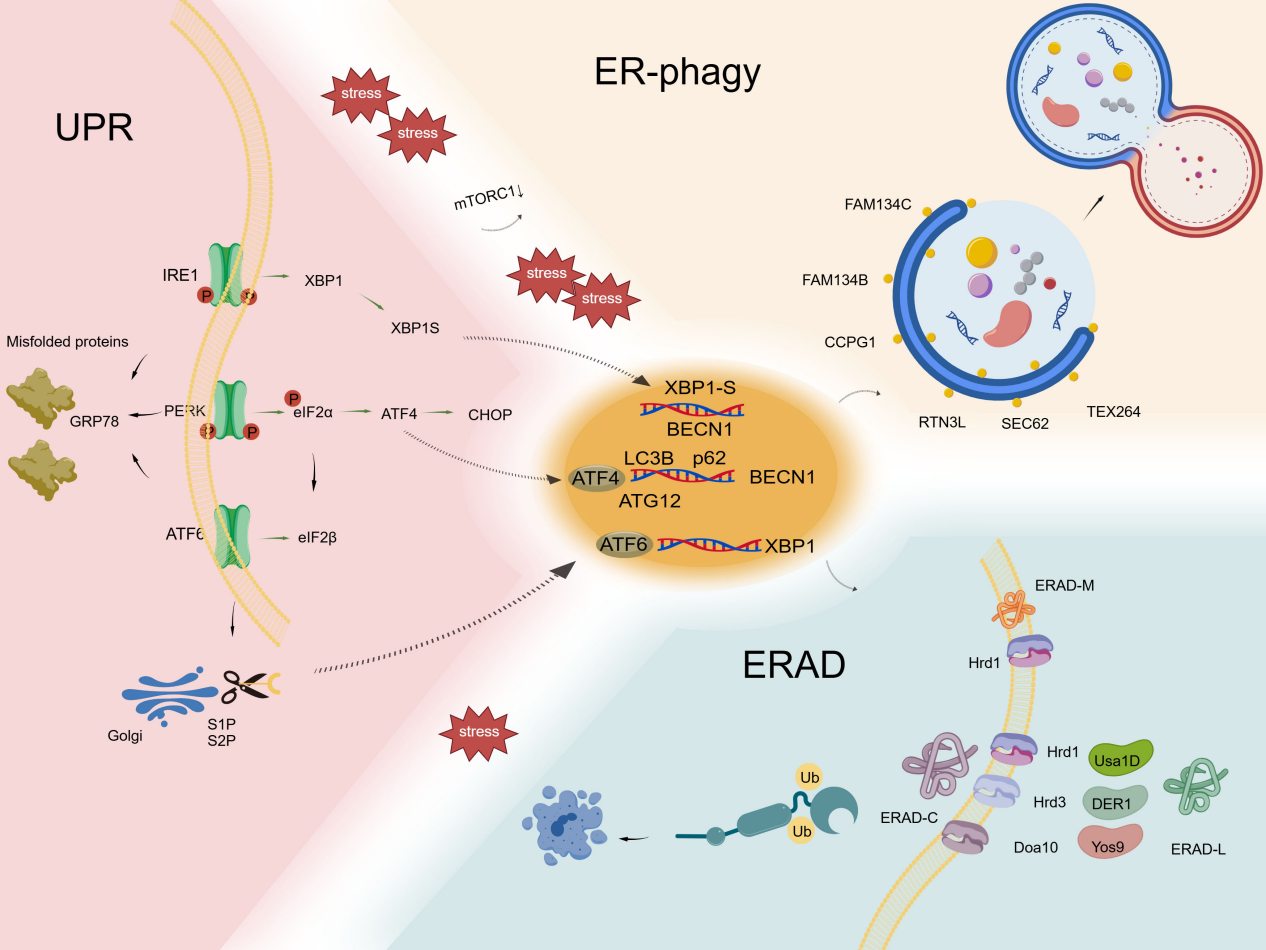
Cooperative regulation of UPR, ERAD, and ER-phagy under ER stress. Under mild stress, the IRE1, PERK, and ATF6 pathways preferentially upregulate ERAD-related molecules to clear misfolded proteins via the proteasome pathway; under strong and persistent stress, UPR pathways induce the expression of ER-phagy receptors and core autophagy components, accompanied by inhibition of mTORC1 signaling, initiating ER-phagy to clear damaged ER fragments. UPR, unfolded protein response; ER, endoplasmic reticulum; ERAD, ER-associated degradation. This image was drawn by BioGDP.com.

In summary, ERAD and the UPR, as core mechanisms of ER quality control, work together to maintain protein homeostasis through multi-level molecular pathways, and their dysregulation is closely associated with the occurrence and development of ER-phagy (see **Figure 1**).

## 3 ER-phagy receptors and regulators

As an important form of selective autophagy, ER-phagy relies on specific proteins to precisely target different ER subdomains to lysosomes for clearance (30). Based on functional characteristics, these proteins can be clearly divided into two categories: classic ER-phagy receptors containing LIR/GIM motifs that directly mediate ER-phagy and regulatory factors that participate in regulating the process but do not act as direct cargo receptors.

### 3.1 Classic ER-phagy receptors

Based on the presence or absence of the reticulon homology domain (RHD), classic ER-phagy receptors are divided into two subcategories. The following sections outline various proteins known to be involved in inflammatory processes (see **Table 1**).

**Table 1:** Overview of ER-phagy receptors.

Protein	Type	Contains RHD	Main localization	Key structure
FAM134B	Classic receptor	Yes	ER sheets	RHD, LIR
FAM134C	Classic receptor	Yes	ER tubules/sheet edges	RHD, LIR
RTN3L	Classic receptor	Yes	ER tubule/endosome/microtubule three-way junctions	RHD, 6 LIR motifs
ATL3	Classic receptor	No	ER	GTPase, GIMs
SEC62	Classic receptor	No	ER	2 transmembrane helices, LIR
CCPG1	Classic receptor	No	Perinuclear ER	LIR, FIR1/2, CIR1/2
TEX264	Classic receptor	No	ER tubule three-way junctions	N-terminal single transmembrane helix, LIR
UBAC2	Classic receptor	No	Perinuclear ER	3 transmembrane domains, LIR, UBA

ER, endoplasmic reticulum; RHD, reticulon homology domain.

#### 3.1.1 Transmembrane ER-phagy receptors containing RHD

##### 3.1.1.1 FAM134B/RETREG1

As the first discovered selective ER-phagy receptor (31), FAM134B is localized to ER sheet-like structures (8). Its core structure is the RHD, which typically contains four transmembrane segments forming two hairpin structures (32). Under ER stress conditions, FAM134B undergoes various post-translational modifications: it can be acetylated at K160 by CREB-binding protein (33) and phosphorylated and activated at S151 by CAMK2β (34). Because FAM134B itself does not enter the ER lumen, it must interact with chaperone proteins that can span the ER membrane, such as the type I transmembrane protein CANX, to target ER-phagy substrates (35). In addition, FAM134B can bind to ARL6IP1 to form a receptor complex, synergistically promoting ER membrane bending and fragmentation. In this process, AMFR further regulates ER remodeling through the ubiquitination of ARL6IP1 and FAM134B (2). Finally, phosphorylation-triggered oligomerization of FAM134B to high-curvature ER membrane regions drives membrane fragmentation and, via its C-terminal LC3- interacting Region (LIR) binding to LC3-labeled autophagosome membranes, initiates the degradation of local ER membranes and associated substrates. FAM134B has two transcripts: the long isoform FAM134B-1, located approximately 9 kbp downstream of the rs257174 block, can be regulated by extracellular ATP (36); the short isoform FAM134B-2, located more than 100 kbp distal, is an N-terminally truncated form that lacks part of the RHD but retains the TM3–4 transmembrane region and the LIR domain (37). It is transcriptionally activated by C/EBPβ under starvation and specifically expressed, not participating in bulk ER degradation but rather mediating the selective degradation of ER-retained secretory proteins (38).

##### 3.1.1.2 FAM134C/RETREG3

Like FAM134B, FAM134C is a transmembrane protein containing the RHD and LIR and controls ER size and morphology via ER-phagy (39). However, unlike FAM134B, which is localized to ER sheet-like structures and responds to ER stress (2), FAM134C is specifically localized to the edges of ER tubules and sheet-like structures, responds to nutrient deprivation, and binds to the ER-localized protein RTN4 (40). Its oligomerization significantly induces ER fragmentation and lysosomal delivery to ensure the clearance of ER subdomains, but it induces ER fragmentation more slowly than FAM134B (41). Moreover, FAM134C exhibits low activity under basal conditions and requires an activation signal for full activation, in contrast to FAM134B, which is fully active under basal conditions (39). Proteomic analysis showed that in cells lacking FAM134B, FAM134A and FAM134C can regulate misfolded procollagen levels normally targeted by FAM134B (39), suggesting functional overlap among the three in some substrate metabolism. One study showed that FAM134A can compensate for the loss of FAM134B and FAM134C in an LIR-independent manner, but FAM134C and FAM134B cannot compensate for each other’s loss (39), indicating a clear difference in substrate recognition breadth among the three. However, a direct association between FAM134A and inflammatory signals has not been confirmed, so we did not discuss FAM134A here.

##### 3.1.1.3 RTN3L

RTN3 has many splice variants, among which the long isoform RTN3L forms discrete punctate structures at the three-way junctions of ER tubules, endosomes, and microtubules (42). Under nutrient-rich conditions, RTN3L interacts via its LIR motif with the FSV region of Rab9a, a key protein in endosomal maturation, and is recruited to ER–endosome membrane contact sites, thereby regulating endosomal maturation and cargo sorting at tubular ER–endomembrane contact sites, whereas nutrient depletion redirects RTN3L to fragmented ER tubules and initiates ER-phagy (42).

Like FAM134B, the RHD of RTN3L consists of two hairpin helices connected by a cytoplasmic linker region, anchoring the protein to the ER membrane and driving ER tubule membrane fragmentation through its oligomerization. Unlike FAM134B, RTN3 contains six LIR motifs in its long N-terminal region and preferentially binds GABARAP; only when all six LIR motifs are mutated is this interaction completely lost (43). Additionally, similar to FAM134B, RTN3L itself does not span the ER membrane (44). Studies have shown that RTN3L can recognize and clear mutant pro-opiomelanocortin by binding to the ER transmembrane protein PGRMC1, while other mutant hormones can be cleared independently of PGRMC1, suggesting the existence of more transmembrane chaperones that cooperate with RTN3L (45).

#### 3.1.2 Transmembrane ER-phagy receptors lacking RHD

##### 3.1.2.1 ATL3

ATL3 is an ER-localized GTPase belonging to the atlastin family (46). When RTN3L is absent, ATL3 acts as the main receptor for tubular ER-phagy (47). It interacts with GABARAP via two GIMs (GABARAP interaction motifs) rather than LC3 (48), specifically promoting the lysosomal degradation of tubular ER fragments containing the tubular ER marker protein REEP5 (49). Furthermore, ATL3 directly interacts with the ULK1 kinase domain and the N-terminal region of ATG13 via its N-terminal GTPase domain, helping to recruit and stabilize ULK1 and ATG101 at the autophagosome formation site defined by the FIP200–ATG13 subcomplex. This action facilitates the initiation of the isolation membrane and the formation of ER-isolation membrane contact sites, providing support for the formation of ER-phagophore (50).

##### 3.1.2.2 SEC62

SEC62 is a 30-kDa integral membrane protein localized to the ER, encoded by a housekeeping gene on chromosome 3q26 (51). It has two transmembrane domains, with both N- and C-termini facing the cytoplasm, a topology crucial for its function (52). In protein homeostasis, SEC62 binds to the C-terminus of SEC63 via electrostatic interactions from positively charged residues in its N-terminal cytoplasmic domain (53), forming a ribosome-free SEC61–SEC62–SEC63 complex (54). This complex mediates the post-translational translocation of secretory proteins into the ER lumen (55); this process can be disrupted by SEC62 deficiency or phosphorylation at Ser341, thereby affecting normal ER degradation (56).

Studies have shown that the loss of Ube2g2 leads to the ineffective degradation of stress chaperones such as Herpud1 and SEL1L, inducing SEC62 upregulation and disrupting the formation of viral replication organelles (57). Upregulated SEC62 binds to lipidated LC3 via its cytoplasmic LIR motif, selectively delivering excess ER components to lysosomes for degradation (58). This process does not depend on autophagosome formation but is driven by the ESCRT-III component CHMP4B and the auxiliary ATPase VPS4A, directly delivering ER-derived vesicles to RAB7/LAMP1-positive endolysosomes for degradation in a form of micro-ER-phagy, effectively restoring ER volume and protein content (59).

##### 3.1.2.3 CCPG1

Unlike the constitutively expressed ER-phagy receptor SEC62, CCPG1 is a type II transmembrane protein localized to the perinuclear ER (60) and responds to ER stress via ERN1–XBP1s–BHLHA14/MIST1 (61). Unlike most other autophagy receptors that only have an LIR motif, CCPG1 has a single LIR and two RB1CC1/FIP200-interacting regions (FIR1/FIR2), which interact with ATG8 family proteins and RB1CC1/FIP200, respectively, initiating ER-phagy (61, 62). In addition, CCPG1 uses its multiple C-terminal cargo-interacting regions (CIRs) to specifically recognize different luminal ER substrates. Among them, CIR1 is required for clearing aggregated proteins, while CIR2 is required for degrading the ER-resident protein P3H4 (prolyl 3-hydroxylase family member 4, non-enzymatic) (63). This mechanism ensures that when CCPG1 is upregulated by ER stress, it can synergistically clear multiple types of damaged components, recruiting various ER-phagy substrates to support restorative autophagy.

##### 3.1.2.4 TEX264

TEX264 (testis-expressed gene 264) is an ER-phagy receptor with a typical type I transmembrane structure. Its hydrophobic and relatively loose N and C termini contain a gyrase inhibitor-like domain that can participate in DNA repair (64). TEX264 is enriched in LC3A/LC3B-positive structures near ER tubule three-way junctions, not only guiding basal ER-phagy but also mediating approximately half of ER-phagy under starvation (65). CK2 kinase phosphorylates serine residues upstream of the LIR motif of TEX264 (65), enhancing its binding affinity to LC3 proteins, thereby initiating ER-phagy (66).

##### 3.1.2.5 UBAC2

Independent of other known ER-phagy receptors, UBAC2 is a transmembrane protein localized to the perinuclear ER. It contains three transmembrane segments, a cytoplasmic LIR motif (sequence WNRL), and a ubiquitin-associated (UBA) domain. Under starvation or ER stress, MARK2 kinase phosphorylates its S223 site, promoting UBAC2 dimerization and subsequent binding to GABARAP, mediating selective autophagic degradation of ER fragments. LIR motif mutation or S223A mutation blocks UBAC2 phosphorylation, dimerization, and GABARAP binding (67, 68). Additionally, UBAC2 can recognize the V-ATPase V1B2 subunit modified by K33-linked polyubiquitination catalyzed by AREL1 via its UBA domain, anchoring lysosomes to the perinuclear ER to maintain their perinuclear localization and normal function (69). Given that the final execution of ER-phagy depends on lysosomes, we speculate that UBAC2-mediated perinuclear anchoring of lysosomes may provide a favorable subcellular microenvironment for efficient ER fragment degradation, but this still awaits further experimental validation.

### 3.2 ER-phagy regulatory factors

#### 3.2.1 CDK5RAP3

As a highly conserved cytoplasmic protein in plants and mammals, CDK5RAP3 (also known as C53/LZAP) is a 506-amino-acid protein with a molecular weight of approximately 66 kDa (70). CDK5RAP3 contains N- and C-terminal alpha-helical domains and an intrinsically disordered region connecting them. This disordered region contains highly conserved shuffled ATG8 interaction motifs (sAIMs) that can simultaneously and competitively bind to UFM1 and the autophagy protein GABARAP, with differences in binding mode and affinity (71). This competitive binding is a key link in the regulation of ER-phagy by the UFMylation system (72).

UFMylation is a ubiquitin-like post-translational modification whose enzymatic cascade is sequentially catalyzed by the E1 enzyme UBA5, the E2 enzyme UFC1, and an E3 ligase complex: UFSP1/UFSP2 first cleave pro-UFM1 to expose C-terminal Gly83; UBA5 activates UFM1 in an ATP-dependent manner and forms a thioester bond; UFM1 is then transferred to Cys116 of UFC1; finally, the E3 complex covalently links UFM1 to lysine residues of substrate proteins (73, 74). The core of this E3 complex is the UFL1–UFBP1–DDRGK1 ternary structure. UFL1 acts as a scaffold-type E3 ligase, with its N-terminus binding UFC1 to receive UFM1 and its C-terminus directly recognizing substrates; UFBP1 anchors the complex to the ER membrane, and its own UFMylation is crucial for stabilizing IRE1α and inhibiting the PERK–CHOP pathway; DDRGK1 recognizes UFM1-modified substrates via its UFIM motif, enhancing substrate capture (74–76).

CDK5RAP3 serves as a substrate adaptor, forming a functional E3 complex with UFL1 and UFBP1. Under resting conditions, the sAIMs of CDK5RAP3 bind to UFM1, shielding their interaction interface with ATG8 family proteins (70); when ER stress leads to ribosome stalling or UFMylation of ribosomal protein RPL26, CYB5R3, etc., UFM1 dissociates from the sAIMs, exposing the sAIMs to bind GABARAP with high affinity, thereby physically connecting the ER subdomain containing UFMylated substrates to the autophagic membrane and initiating ER-phagy (77–79). Under ER stress conditions, this pathway exhibits dynamic bidirectional regulation: on the one hand, stress signals through the transcription factor XBP1 upregulate the expression of UFMylation system components, forming a positive feedback adaptation loop (80). On the other hand, stress-induced ribosome stalling directly enhances RPL26 UFMylation, especially CDK5RAP3-dependent dual modification at K132, promoting 60S ribosomal subunit dissociation from the SEC61 translocon, while CDK5RAP3 recruits GABARAP to initiate ER-phagy, synergistically clearing stalled nascent chains and damaged ER fragments (75, 81). When CDK5RAP3 deficiency leads to UFMylation dysfunction, IRE1α stability decreases, PERK–CHOP is abnormally activated, ER stress intensifies, and ER-phagy is blocked, ultimately shifting cells from adaptive protection to apoptosis (74, 82). In summary, CDK5RAP3, through its sAIMs, acts as a “UFMylation status sensor” and is a key molecular switch for maintaining protein homeostasis and determining cell fate under ER stress.

#### 3.2.2 ARL6IP1

ARL6IP1 is an ER membrane-shaping protein simultaneously localized to smooth ER tubules and mitochondria-associated ER membranes (MAMs) (83). Its RHD consists of two long hydrophobic regions, each of which does not span the membrane but forms short hairpin conformations embedded in the outer leaflet of the lipid bilayer, thereby inducing membrane curvature. Functional studies have shown that the overexpression of ARL6IP1 significantly induces the widespread formation of peripheral ER tubules and causes strong ER membrane contraction, excluding luminal proteins (such as PDI) from peripheral ER tubules. Further analysis has shown that ARL6IP1 forms oligomers on the ER membrane, and its overexpression is sufficient to maintain ER tubule network structure under microtubule depolymerization conditions. These results suggest that ARL6IP1, as a microtubule-independent ER membrane-shaping protein, can actively drive high curvature and stable tubule formation of the ER membrane (84, 85).

In terms of molecular mechanism, ARL6IP1 itself does not bind LC3 and cannot independently act as an ER-phagy receptor. However, it can form heterologous complexes with another RHD protein, FAM134B, co-localizing to the same ER regions and thereby enhancing FAM134B-mediated ER-phagy. This interaction depends on the central RHD-containing region of both proteins (2, 84). Similar phenomena exist for FAM134C and RTN4, but whether FAM134A, which also contains an RHD, has the same effect remains to be determined (2).

#### 3.2.3 AMFR

AMFR (also known as gp78) is an ER-integral membrane protein whose gene is located on human chromosome 16 with a molecular weight of approximately 78 kDa (86). The cytoplasmic C-terminal region of AMFR (residues 309–643) constitutes an approximately 63-kDa E3 ubiquitin ligase active domain containing a conserved RING domain, giving it the ability to catalyze substrate ubiquitination (86, 87).

In the regulation of ER-phagy, AMFR plays a key role. Its substrates include not only the classic ER-phagy receptor FAM134B but also the ER membrane-shaping protein ARL6IP1. Studies have shown that AMFR, as an E3 ligase, catalyzes the ubiquitination of FAM134B, which promotes FAM134B aggregation, thereby driving ER membrane remodeling and enhancing ER-phagy flux (88). At the same time, AMFR also catalyzes the ubiquitination of ARL6IP1 at key sites K96 and K114, making ARL6IP1 more compact and enhancing its membrane-shaping ability, while promoting the binding of the FAM134B–ARL6IP1 heterodimer to LC3B; a ubiquitination-deficient ARL6IP1-7KR mutant shows significantly reduced LC3B binding (2, 85). In addition, the deubiquitinating enzyme USP20 stabilizes FAM134B by removing K48- and K63-linked ubiquitin chains from FAM134B, cooperating with AMFR to promote ER-phagy (88).

## 4 Regulatory mechanisms of ER-phagy

In fact, the initiation of ER-phagy is a multi-signal integration process that goes beyond the classic UPR and ERAD pathways. Among them, AMPK, mTORC1, and CK2, as core sensors of cellular metabolism, constitute the hub of the regulatory network; however, ROS and Ca^2+^ homeostasis imbalance act as “danger signals” of ER damage, working together to determine the initiation and intensity of ER-phagy.

### 4.1 Metabolic sensing and nutrient signaling pathways regulating ER-phagy

The expression and activity of ER-phagy receptors are directly regulated by cellular energy status and nutrient supply. AMPK, mTORC1, and CK2 are three key regulatory kinases that, through phosphorylation, regulate the activity of ER-phagy receptors and their complexes, synergistically responding to nutrient and energy status to determine the initiation or inhibition of ER-phagy (89–91).

#### 4.1.1 AMPK

As the core sensor of cellular energy metabolism, AMPK is activated upon phosphorylation at Thr172. Activated AMPK promotes ER-phagy mainly through two classic pathways: one is by inhibiting mTORC1 activity, relieving its basal inhibition of ER-phagy, and the other is by directly phosphorylating and activating the ULK1/2 complex. Both pathways together induce the occurrence of ER-phagy (92). In addition, studies have shown that non-canonical ER-phagy mechanisms independent of the classic AMPK-mTORC1 and AMPK-ULK1 pathways also exist, suggesting the diversity and cell-type dependence of the regulatory network (93, 94). Notably, AMPK activity itself is also affected by intracellular ROS levels: low concentrations of ROS activate AMPK through oxidative modification, while high concentrations of ROS may indirectly activate AMPK by excessive ATP consumption, forming a combination of oxidation, energy, and stress (95).

#### 4.1.2 mTORC1

mTORC1, a member of the PI3K-related kinase family, is a serine/threonine kinase composed of mTOR, Raptor, mLST8/GβL, and DEPTOR. It can bidirectionally regulate ER-phagy by sensing the nutritional status of the body (90). Under nutrient-rich conditions, mTORC1 is recruited to the lysosomal surface and activated. Activated mTORC1 phosphorylates ULK1 and inhibits its activity, blocking the basal occurrence of ER-phagy; under starvation, its activity is inhibited, relieving the inhibition of ULK1 and promoting TFEB/TFE3 nuclear translocation, thereby bidirectionally initiating ER-phagy receptors (96). Notably, under basal conditions, mTORC1 has a certain regulatory effect on the expression of the ER-phagy receptor FAM134B-2, but under amino acid deficiency, FAM134B-2 is regulated by the MEF2D–NR4A1 pathway (97), indicating that cells can selectively activate corresponding modules under different physiological or stress conditions to precisely maintain ER homeostasis.

However, the response of mTORC1 to nutritional signals does not occur in isolation; its activity can be regulated by Ca^2+^ signals. An increase in cytoplasmic Ca^2+^ concentration can activate CaMKK2, which in turn phosphorylates and activates AMPK, ultimately achieving inhibition of mTORC1. This indicates that Ca^2+^ signals can trigger ER-phagy even under nutrient-rich conditions by hijacking the energy-sensing pathway (98). In addition, under starvation conditions, the inhibition of mTORC1 reduces the phosphorylation level of FAM134C-LIR at S435, S436, and T440 by CK2, suggesting that mTORC1 may indirectly affect CK2-mediated phosphorylation regulation (91).

#### 4.1.3 CSNK2/CK2

CSNK2/CK2 is a serine/threonine kinase composed of catalytic subunits (α/α′) and regulatory subunits (β). It regulates ER-phagy by phosphorylating different sites on ER-phagy receptors. Under starvation, CK2 phosphorylates S149, S151, and S153 of the FAM134B-RHD and S258 and S260 of the FAM134C-RHD, promoting the functional ubiquitination and subsequent clustering and high-density cluster formation of these receptors (99, 100). In addition, CK2 can phosphorylate S271 and S272 of TEX264-LIR, forming specific hydrogen bonds that enhance its binding affinity to LC3/GABARAP, thereby promoting ER-phagy under starvation (101). Notably, under starvation, CK2 phosphorylation levels at S435, S436, and T440 near the FAM134C-LIR are reduced, thereby enhancing its binding to LC3B and promoting ER-phagy (91, 102). This opposite effect originates from the nature of the phosphorylation sites and their position relative to the LIR domain: the inhibitory phosphorylation sites of FAM134C are adjacent to the LIR, and CK2 promotes FAM134C-LC3B binding and ER-phagy by reducing the phosphorylation level at these sites; the enhancing phosphorylation sites of TEX264 are located adjacent to the LIR, and CK2 promotes TEX264-LC3B binding and ER-phagy by increasing the phosphorylation level at these sites (101).

### 4.2 MAMs integrate Ca^2+^-ROS signaling and the initiation of ER-phagy

The IP3R–GRP75–VDAC1 complex is the core channel for Ca^2+^ transport on MAMs, and its integrity is crucial for autophagosome formation (103, 104). Under physiological conditions, PARKIN is recruited to MAMs by IP3R-mediated Ca^2+^ flux and then marks IP3R via K48-linked ubiquitination for proteasomal degradation, thereby limiting IP3R accumulation in MAMs and preventing excessive Ca^2+^ efflux from triggering excessive ER stress (105). Under stress conditions, the IP3R–GRP75–VDAC1 complex mediates enhanced Ca^2+^ release, which enters mitochondria via VDAC1 and MCU, leading to Ca^2+^ overload (106, 107). In addition, ROS generated by the large amount of misfolded proteins induced by stress further oxidatively activate IP3R, exacerbating Ca^2+^ release (108–110); mitochondrial Ca^2+^ overload then activates TCA dehydrogenases and NOS, promotes QH accumulation, and induces mPTP opening to release cyt c, mtDNA, etc., inhibiting complex III and glutathione defense and further driving ROS production, thereby exacerbating ER stress (109, 110). At the early stage of stress or the initiation phase of autophagy, PINK1 and BECN1 relocate to MAMs and, through interaction with the IP3R–GRP75–VDAC1 complex, enhance MAMs contact and promote omegasome formation (111). When stress continues to intensify, mitochondrial Ca^2+^ overload causes membrane potential drop and ATP depletion, weakening the energy base for autophagy; at the same time, it upregulates DRP1/FIS1 and downregulates MFN2/OPA1, promoting fission and inhibiting fusion, leading to mitochondrial fragmentation, which in turn destroys MAM structure and function, reducing the membrane source for autophagosomes (112, 113). Notably, sustained stress leads to downregulation of the MAM structural protein MFN2, which inhibits GRP75–VDAC1 binding, reduces mitochondrial Ca^2+^ uptake, and significantly downregulates the expression of autophagy proteins such as LC3-II, Atg5, and Atg7, thereby inhibiting autophagosome formation (114).

**Figure 2:**
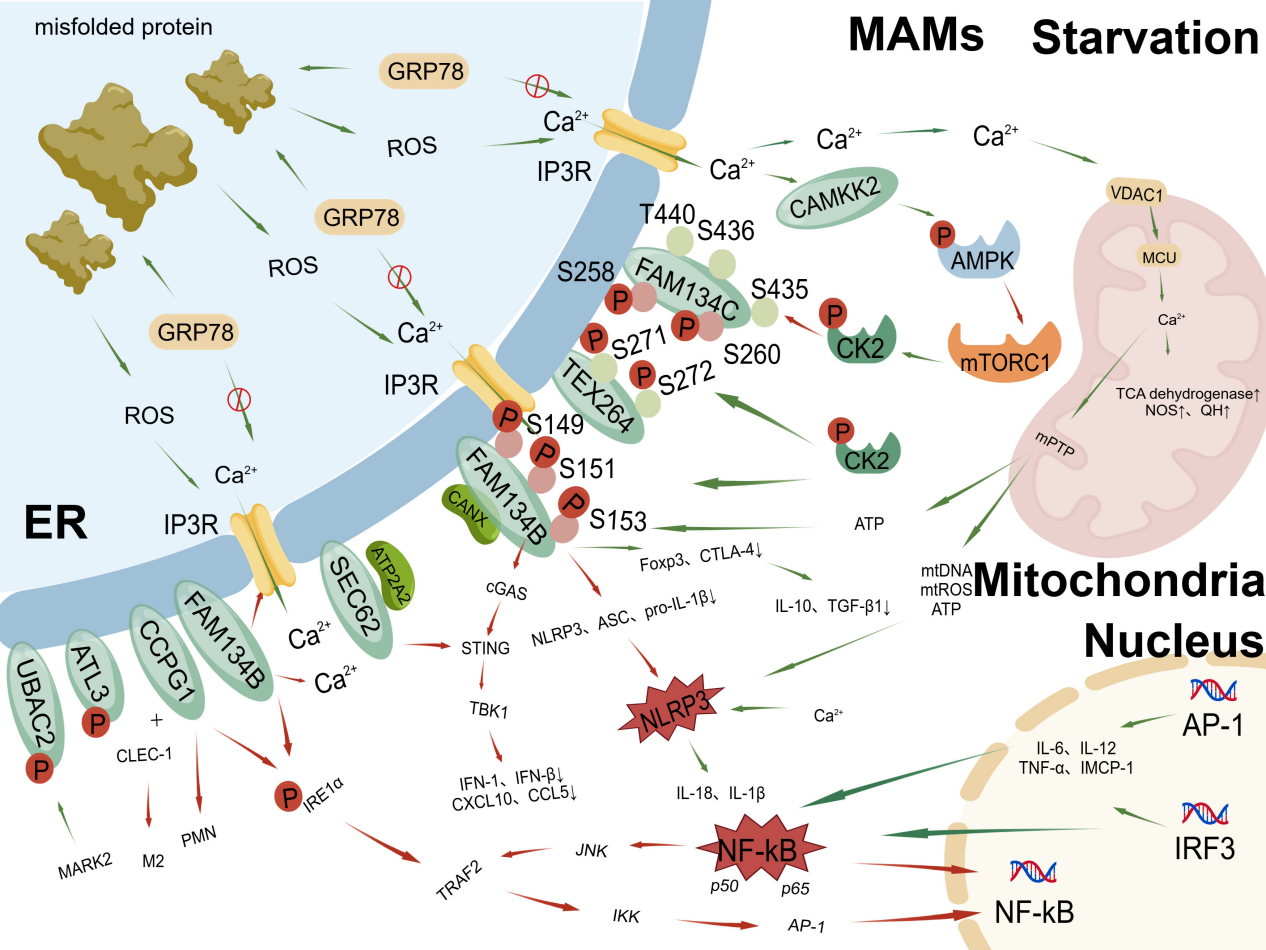
ER-phagy regulatory network and its negative regulatory mechanism in inflammatory pathways. Under ER stress, IP3R-mediated Ca^2+^ efflux forms a positive feedback with ROS, activating the AMPK–CK2 signaling axis to promote ER-phagy mediated by receptors such as FAM134B/C and TEX264. On the one hand, ER-phagy restores Ca^2+^-ROS homeostasis by clearing damaged ER fragments and inhibiting ER–mitochondrial calcium transport. On the other hand, it reduces pro-inflammatory factor release by targeting and inhibiting the cGAS–STING, NF-κB, and NLRP3 inflammatory pathways, thereby avoiding excessive inflammatory damage. ER, endoplasmic reticulum. This image was drawn by BioGDP.com.

To cope with sustained stress and ER Ca^2+^ and redox homeostasis imbalance, cells initiate ER-phagy, a selective autophagy pathway, to actively clear damaged ER fragments and restore Ca^2+^ and ROS homeostasis. Specifically, ER-phagy, on the one hand, upregulates ER-phagy receptors such as FAM134B, TEX264, UBAC2, and SEC62 to directly clear calcium store-abnormal and oxidatively damaged ER fragments, indirectly restoring Ca^2+^ and ROS homeostasis (115–118). On the other hand, MAMs are not only a Ca^2+^ transport hub but also a potential platform for initiating ER-phagy (119, 120). ER-phagy receptors also play key roles in this process: for example, FAM134B restricts abnormal Ca^2+^ exchange by clearing MAM regions containing excess IP3R in epileptic neurons (121); FAM134B interacts with the MAM-resident protein CANX to promote ER fragmentation and autophagic engulfment (111). ATL3 maintains MAM structural integrity and limits excessive Ca^2+^ transfer from the ER to mitochondria (122), suggesting that ER-phagy is no longer just a passive responder to damage but an active participant in maintaining and repairing cellular homeostasis (see **Figure 2**).

## 5 ER-phagy in inflammatory responses

### 5.1 Inhibitory effect of ER-phagy on the cGAS–STING signaling pathway

As a key DNA sensor, STING responds to exogenous DNA to initiate pro-inflammatory responses (123) and can also be activated by cGAS due to impaired clearance of endogenous DNA (124). After the activation of the cGAS–STING pathway, STING translocates from the ER to the Golgi apparatus; on the one hand, it activates TBK1 to phosphorylate IRF3, inducing IFN-I gene transcription. On the other hand, it activates the IKK–NF-κB axis to promote the release of pro-inflammatory factors, such as IL-6 and TNF-α, and simultaneously triggers NLRP3 inflammasome maturation by inducing lysosomal membrane permeabilization and K^+^ efflux, thereby releasing IL-1β and IL-18 (125). However, in cancer cells, damaged DNA activates the caspase pathway, which in turn induces IL-33 and CXCL10 expression. Tumor cells secrete CCL5 and CXCL10 and produce IFN-β, collectively recruiting and activating NK cells; activated NK cells further secrete IFN-γ, granzyme B, and perforin to synergistically inhibit tumor progression (126). In addition, cGAS recognizes cytoplasmic DNA and catalyzes the production of cGAMP, which activates STING; downstream, the TBK1-IRF3 and NF-κB signaling axes induce the expression of SASP factors. This pathway can recruit immune cells to clear precancerous cells in the short term, exerting anti-tumor effects; but long-term persistent activation upregulates COX-2 and promotes PGE2 synthesis, inhibiting anti-tumor immunity and driving chronic inflammatory microenvironment remodeling, ultimately promoting tumor occurrence and progression (127).

Notably, some ER-phagy receptors have been found to negatively regulate inflammatory signals by modulating cGAS–STING pathway activity. Among them, significant upregulation of FAM134B can mediate the degradation of cGAS–STING-TBK1 pathway proteins and downstream TBK1 and IRF3 via the autophagic pathway, blocking IFN-I production and indirectly inhibiting NLRP3 inflammasome assembly and activation by clearing abnormally accumulated proteins and damaged ER, thereby reducing the maturation and release of downstream pro-inflammatory factors IL-1β and IL-18 (128). In contrast to the multi-target regulatory mode of FAM134B, SEC62 requires the guidance of ATP2A2 to bind to activated STING1, forming the “SEC62–STING1–ATP2A2” complex. This complex targets STING1 for autophagic–lysosomal degradation by promoting LC3B lipidation and autophagosome formation, thereby terminating STING1 signaling and preventing excessive release of IFN-β and inflammatory factors such as CXCL10 and CCL5 (129).

### 5.2 Inhibitory effect of ER-phagy on the NF-κB signaling pathway

NF-κB is a transcription factor complex composed of p50, p52, p65(RelA), c-Rel, and RelB subunits. Its activation is crucial for initiating pro-IL-1β production and NLRP3 transcription (130). In the resting state, NF-κB is bound to the inhibitory protein I-κB and retained in the cytoplasm in an inactive form; its classic activation pathway begins with I-κB kinase-mediated I-κB phosphorylation and ubiquitin–proteasome degradation, thereby releasing NF-κB and allowing its nuclear translocation (131).

The ER can activate NF-κB through multiple mechanisms. On the one hand, the ER membrane E3 ligase HRD1 inhibits the deubiquitinating enzyme Usp15, preventing it from deubiquitinating IκBα, thereby promoting IκBα degradation and activating NF-κB (132). On the other hand, ER stress participates in regulating NF-κB activation through multiple branches. Upon activation, ATF6 upregulates CHOP and activates NF-κB via the TLR-MyD88 pathway (133, 134); meanwhile, the PERK–eIF2α–ATF4 axis can induce TRAIL receptor expression, recruiting FADD, caspase-8, and RIPK1 to assemble into a “FADDosome” complex (135), which synergizes with the IRE1α–TRAF2–ASK1–JNK pathway to amplify NF-κB signaling. In addition, sustained ER stress leads to massive Ca^2+^ leakage into the cytoplasm, activating CaM and CaMKII (136, 137). Among these, the atypical PKC isoform of CaM cooperates with excess ROS to regulate IKK and promote IκBα degradation, thereby driving nuclear transcription of NF-κB subunits p65/p50 and cRel; however, CaMKII mainly acts downstream of the NF-κB signaling pathway, enhancing the transcriptional activity of nuclear NF-κB by phosphorylating the transcriptional coactivator CBP/p300, without affecting its nuclear localization (138, 139).

Notably, the ER-phagy receptor CDK5RAP3 is a negative regulator of NF-κB. On the one hand, CDK5RAP3 directly binds to the RelA subunit, affecting its Ser536 phosphorylation. On the other hand, CDK5RAP3 enhances the binding of RelA to histone deacetylases HDAC1, HDAC2, and HDAC3, promoting the deacetylation of chromatin regions occupied by RelA, thereby inhibiting NF-κB transcriptional activity and downregulating the expression of various NF-κB target genes such as MMP-9, IL-8, and IL-6 (140). The ER-phagy receptor FAM134B-mediated ER-phagy inhibits NF-κB signaling activation by clearing damaged or excessively expanded ER and reducing ER stress. Its mechanism may involve that FAM134B-mediated ER-phagy clears damaged or stressed ER fragments and reduces misfolded protein accumulation in the ER lumen, thereby inhibiting the overactivation of upstream stress sensors such as IRE1α, reducing the subsequent recruitment and activation of IKK by TRAF2, decreasing IκB phosphorylation and p65 nuclear translocation, and ultimately inhibiting NF-κB and its mediated transcription of pro-inflammatory factors such as IL-6, IL-1β, and TNF-α (141).

### 5.3 Inhibitory effect of ER-phagy on the NLRP3 inflammasome

As an important member of the NLR family, the NLRP3 inflammasome activation depends on a “two-signal” process: the first priming signal is the NF-κB pathway-mediated upregulation of NLRP3, pro-caspase-1, and pro-IL-1β expression; the second signal is NLRP3 oligomerization induced by Ca^2+^, K^+^, ROS, etc., recruiting ASC and pro-caspase-1 to form an activation complex. Activated caspase-1 cleaves GSDMD to generate membrane pores and promotes the maturation and release of IL-1β or IL-18 (142, 143). Specifically, in the second activation signal, Ca^2+^ occupies a central hub position. On the one hand, Ca^2+^ itself can directly synergize with the first signal to promote NLRP3 oligomerization and ASC speck formation (144). On the other hand, Ca^2+^ activates G protein-coupled calcium-sensing receptors such as CaSR and GPRC6A on the membrane and, via PLC-mediated intracellular Ca^2+^ release and cAMP decrease, drives caspase-1 activation and IL-1β release (145). More importantly, Ca^2+^ and ROS form a mutually amplifying positive feedback loop. Ca^2+^ can promote ROS generation by activating CaMKII and other kinases (146); ROS, in turn, can activate TRPM2 and BK channels by promoting ADPR production, leading to Ca^2+^ influx and K^+^ efflux, further amplifying the activation signal (147, 148). When large amounts of Ca^2+^ enter mitochondria, they can induce mitochondrial membrane potential depolarization and dysfunction and induce mtROS burst, and the accumulated ROS can directly oxidatively modify NLRP3 protein or upregulate adaptor molecules such as TXNIP to bind NLRP3, ultimately promoting full NLRP3 inflammasome assembly and activation of downstream caspase-1 and cytokine release (149, 150).

The ER, as the core organelle maintaining Ca^2+^ and ROS homeostasis, negatively regulates NLRP3 inflammasome activation through multiple pathways via its autophagic process. On the one hand, ER-phagy indirectly maintains the function of TRPML1, TMEM175, and BK ion channels by clearing abnormal ER, thereby maintaining lysosomal membrane potential and pH homeostasis and inhibiting lysosomal membrane permeabilization, cathepsin B release, K^+^ efflux, and ROS elevation that activate NLRP3 (151). On the other hand, studies have shown that ER-phagy maintains the structural integrity of MAMs, regulating Ca^2+^ transfer to mitochondria mediated by the IP3R–GRP75–VDAC1–MCU complex, which helps inhibit mtROS and mtDNA generation and mitochondrial damage caused by Ca^2+^ overload, thereby inhibiting NLRP3 inflammasome assembly at MAMs (104). In addition, the FAM134B-mediated ER-phagy receptor inhibits NLRP3 activation by clearing damaged ER and alleviating ER stress (152). Its specific molecular mechanisms may include two aspects: on the one hand, FAM134B-mediated ER-phagy clears misfolded proteins or Ca^2+^ accumulated on the ER, inhibiting inflammasome activation signals at the source. On the other hand, FAM134B-mediated ER-phagy can degrade key components of the NLRP3 inflammasome, such as NLRP3, ASC, and pro-IL-1β, thereby inhibiting the excessive activation of the NLRP3 inflammasome.

### 5.4 Role of ER-phagy in maintaining immune cell homeostasis

As the first line of defense against pathogen invasion, dysfunction of neutrophils directly exacerbates inflammation, and this process is closely related to ER-phagy dysregulation. In neutrophils, FOXO3 deficiency leads to downregulation of CCPG1 transcription, reducing the ability to clear ER stress; persistent ER stress activates the NF-κB pathway via the IRE1α–TRAF2 complex, promoting the expression of pro-inflammatory factors such as IL-1β, IL-6, and TNF-α, while also enhancing neutrophil chemotaxis and migration, causing massive infiltration into colonic tissues and release of ROS and proteases, disrupting the intestinal epithelial barrier, and promoting colitis-associated tumorigenesis (153).

Second, the resolution of inflammation depends on the subsequent polarization conversion of macrophages from pro-inflammatory M1 to anti-inflammatory M2, as well as the clearance of apoptotic neutrophils by M2 macrophages. During corneal *Aspergillus fumigatus* infection, CCPG1 expression increases and co-localizes with the inhibitory receptor CLEC-1 in macrophages, suggesting that they may synergistically regulate macrophage function in inflammatory responses, but the specific interaction remains to be studied (154). In tumor-associated macrophages (TAMs), CDK5RAP3 inhibits NF-κB p65 phosphorylation and its nuclear transcription, thereby reducing the secretion of downstream M2 polarization-related cytokines IL-4 and IL-10, inhibiting TAM polarization toward the M2 phenotype, and promoting M1 polarization, while also inhibiting the CCL2/CCR2 signaling axis and reducing circulating monocyte recruitment to tumor tissues, thereby reshaping the anti-tumor immune microenvironment (155).

As the effector and regulatory center of adaptive immune responses, the dynamic balance between effector T cells and regulatory T cells (Tregs) determines the direction and intensity of inflammation (156). Among them, Tregs are core cells that maintain immune tolerance, inhibiting the activity of effector T cells, dendritic cells, and macrophages by secreting IL-10, TGF-β, and expressing CTLA-4, thereby maintaining immune tolerance; their functional or numerical abnormalities can lead to chronic inflammation and autoimmunity (157). Studies have shown that IL-36β upregulates the expression of the ER-phagy receptor FAM134B by activating the PERK–ATF4 signaling pathway, thereby inducing ER-phagy in CD4+CD25+ regulatory T cells, causing decreased expression of key immunosuppressive molecules Foxp3 and CTLA-4, reduced secretion of anti-inflammatory cytokines IL-10 and TGF-β1, and impaired ability to inhibit effector T-cell proliferation, thereby alleviating excessive immunosuppression in sepsis (17). In addition, ATP, as a pro-inflammatory molecule, upregulates the expression of the long isoform of FAM134B in monocytes in a dose-dependent manner; CD39 on the surface of Tregs hydrolyzes extracellular ATP, indirectly inhibiting ATP-induced FAM134B expression, forming a transcellular genetic regulation mechanism involved in the pathogenesis of allergic diseases (36).

### 5.5 Interaction between ER-phagy and immune-related phagocytic pathways

In recent years, the interaction between ER-phagy and various immune-related phagocytic pathways has attracted increasing attention, mainly manifested in its synergistic regulation with general autophagy, LC3-associated phagocytosis (LAP), and conventional phagocytosis.

ER-phagy and general autophagy form functional complementarity under immune-related stress conditions. For example, in sepsis, FAM134B-mediated ERGICphagy selectively clears STING1 aggregates on the ERGIC, inhibiting the CASP3–GSDME pyroptosis pathway; however,while general autophagy non-selectively degrades ERGIC and STING1 itself, with the two responsible for selective clearance of activation platforms and broad-spectrum degradation of signaling molecules, respectively, jointly maintaining dendritic cell immune homeostasis (8). In macrophages stimulated with magnetic iron oxide nanoparticles, ER stress via the IRE1α–CHOP axis induces both general autophagy mechanisms and ER-phagy; ER-phagy depends on the core molecules of general autophagy, LC3, and lysosomal acidification, to clear stressed ER, thereby alleviating ER stress and inhibiting apoptosis (158). In antiviral immunity, irreversible ER stress upregulates general autophagy via the PERK–eIF2α–ATF4 pathway to promote cell death while specifically inducing FAM134B expression to drive ER-phagy to degrade STING protein on the ER membrane, leading to downregulation of IRF9 and p-STAT1, thereby weakening the cGAS–STING-mediated antiviral response, and both synergistically promote viral replication (159). In summary, ER-phagy and general autophagy can either divide labor complementarily or be interdependent or synergistically regulate immune responses under various stress conditions, forming a complex interactive regulatory network.

In the interaction between ER-phagy and LAP, both share a non-canonical LC3 lipidation pathway and molecular regulatory nodes. First, in host anti-infection defense, STING, upon activation, directly binds LC3 family proteins via its LIR motif and induces LC3 lipidation onto ER-derived single-membrane vesicles in a V-ATPase- and ATG16L1-dependent manner, but independent of classic autophagy initiation factors such as ULK1 and VPS34; these vesicles fuse with lysosomes to clear bacterial components and damaged ER, a mechanism that shares the non-canonical LC3 lipidation pathway with LAP (160, 161). Second, the ER-phagy receptor FAM134B also utilizes this pathway, binding to lipidated LC3/GABARAP via its LIR motif to mediate ER fragment encapsulation and clearance of viruses such as Ebola and flaviviruses (160). However, some viruses have evolved counter-strategies: coronaviruses hijack LC3-positive EDEMosomes (ER-derived vesicles generated by the ERAD pathway) as replication platforms, a process that belongs to the LAP-like single-membrane LC3 lipidation pathway (161). In addition, the ER supports LAP function via Ca^2+^ signaling: as an intracellular Ca^2+^ store, the ER releases Ca^2+^ through ER–phagosome membrane contact sites, activating calmodulin signaling, which in turn promotes Rubicon recruitment, VPS34 activation, PI(3)P generation, NOX2 assembly, and LC3 lipidation, ultimately driving LAPosome formation, playing a key role in antifungal immunity (162).

In immune defense, conventional phagocytosis is responsible for pathogen engulfment and antigen presentation, while the ER provides a key membrane source for phagosomes through membrane contact, calcium signaling regulation, and membrane fusion, supporting the formation of LAP: LC3 is recruited to the phagosomal membrane, accelerating phagosome maturation, promoting apoptotic cell clearance, and efficiently mediating antigen cross-presentation in dendritic cells; at the same time, ER–phagosome fusion provides a dedicated subcellular platform for cross-presentation of exogenous antigens on MHC class I molecules, bridging innate and adaptive immunity (163–166). However, ER-phagy inhibits the unfolded protein response and sterile inflammation by degrading damaged ER fragments, and its receptors can directly encapsulate and clear ER structures used for virus replication, enhancing host antiviral capacity (167). In summary, both share core molecular mechanisms such as LC3 and synergistically complement each other in phagosome–ER interaction, antigen processing, anti-microbial immunity, and inflammation regulation, jointly forming a complete immune defense network from exogenous pathogen clearance to intrinsic quality control.

## 6 ER-phagy in inflammatory diseases

### 6.1 Infection

The ER lumen is a site for replication and assembly of various intracellular bacteria and enveloped viruses (168). ER-phagy plays a dual role in infection. On the one hand, ER-phagy can eliminate invading pathogens by degrading damaged ER, participating in host defense (41, 169). Taking Gram-positive bacteria as an example, the cyclic di-nucleotide c-di-AMP they release is recognized by STING, which then activates the PERK/IRE1α–CHOP pathway and inhibits mTORC1, thereby inducing ER-phagy. This process not only alleviates ER stress but also promotes the binding of STING and TBK1 on autophagosomes and enhances the production of IFN-I, strengthening the anti-inflammatory response (170).

On the other hand, many pathogens have evolved immune evasion strategies that specifically cleave or modify proteins to disrupt host ER-phagy homeostasis, achieving immune escape and further exacerbating viral pathogenicity (169). For example, the *Salmonella* effector protein SopF reduces the phosphorylation of FAM134B at S151 and acetylation at K160, inhibiting RHD-mediated oligomerization, thereby blocking ER membrane cleavage and autophagosome wrapping and effectively blocking ER-phagy to evade host immune clearance and exacerbate inflammatory responses (34). Viruses have even more diverse mechanisms to hijack ER-phagy. For instance, the protease complex NS2B3 of flaviviruses such as dengue and Zika viruses directly cleaves FAM134B at the non-classical site se142 within the RHD, generating a C-terminal fragment that cannot oligomerize; although this fragment still contains the LIR motif, it loses the ability to induce ER membrane curvature, thereby blocking ER-phagy and subsequent viral degradation (171). SARS-CoV-2 non-structural protein 6, on the one hand, activates FAM134B- and CCPG1-mediated ER-phagy to selectively degrade key proteins of the RLR pathway and inhibit cGAS–STING immune signaling, thus suppressing downstream IFN production. On the other hand, it binds to the ER-resident protein HSPA5/GRP78, blocking its interaction with EIF2AK3/PERK, thereby specifically activating the EIF2AK3–EIF2A signaling pathway, inducing strong ER stress, upregulating pro-inflammatory factors such as IL-6 and TNF, and creating an environment favorable for viral replication and harmful inflammation, thereby antagonizing the host’s antiviral innate immunity (10). Notably, AMFR-mediated ubiquitination can also be hijacked by viruses: the flavivirus NS2A protein localizes to the ER and is ubiquitinated by AMFR with K48-linked polyubiquitination, and then ubiquitinated NS2A binds to FAM134B, promoting the degradation of this complex, thereby inhibiting FAM134B-driven ER-phagy, leading to ER accumulation and exacerbating viral pathogenicity (172). Furthermore, other viruses promote the interaction between ATL3 and ARF4 to regulate early endosome trafficking, ensuring that furin can recycle to the viral assembly site to complete prM cleavage and maturation, assisting in the maturation and release of viral particles (173).

### 6.2 Sepsis

Sepsis is a multi-organ dysfunction syndrome with poor prognosis, characterized by an initial high inflammatory response after infection, followed by a more persistent immunosuppressive phase (174). Therefore, restoring the balance of the host inflammatory response is of utmost importance in the treatment of sepsis.

As a key link in sepsis-related multi-organ dysfunction, cardiac dysfunction is caused by vascular leakage due to excessive production of inflammatory factors such as TNF-α, IL-6, and IL-8 (175). FAM134B-mediated ER-phagy reduces the release of pro-inflammatory factors such as TNF-α, IL-6, and IL-8 and increases the level of the anti-inflammatory factor IL-10 by inhibiting the activation of IRE1α and thereby blocking the downstream NF-κB signaling pathway, while also reducing cardiomyocyte apoptosis-related proteins, cleaved caspase-3, and BAX, and increasing the anti-apoptotic protein BCL-2, ultimately alleviating myocardial tissue inflammatory damage and apoptosis and improving sepsis-induced cardiac dysfunction (176).

Furthermore, in sepsis-induced intestinal barrier injury, the nuclear receptor Nur77 is activated. Activated Nur77, on the one hand, directly binds to NLRP3 and migrates to the trans-Golgi region, competitively occupying the dispersed trans-Golgi network platform required for NLRP3 inflammasome assembly, thereby blocking NLRP3-ASC assembly and subsequent inflammasome activation (177). On the other hand, Nur77 interacts with PKCα and translocates to the ER, promoting the phosphorylation of AMFR, which in turn catalyzes the ubiquitination of FAM134B, activating FAM134B-mediated ER-phagy and maintaining ER homeostasis by clearing damaged or excessive ER. This process inhibits Paneth cell necroptosis and reduces the release of pro-inflammatory mediators such as TNF-α and HMGB1, thereby alleviating local macrophage infiltration and intestinal epithelial barrier damage and mitigating sepsis-related intestinal inflammation (12).

### 6.3 Digestive system diseases

In inflammatory diseases of the digestive system, ER-phagy generally plays a protective role, and its functional loss or deletion of regulatory factors aggravates the pathological process, with this pattern showing organ-specific manifestations in the liver, pancreas, and intestine.

As the core organ for digestion and metabolism, the liver’s inflammatory responses are closely related to ER-phagy. In acute liver injury caused by ischemia–reperfusion, the ER membrane calcium channel protein GRINA directly binds to ATF6 and recruits the E3 ubiquitin ligase HRD1 to form a complex; through HRD1-mediated ubiquitination, it regulates ATF6 activation and nuclear translocation, thereby upregulating ER-phagy receptors and pathway activity, initiating ER-phagy to clear damaged ER, inhibiting ER stress-induced NF-κB activation, reducing the release of inflammatory factors (such as TNF-α, IL-1β, IL-6, and MCP-1), and alleviating liver inflammatory damage (21). In chronic liver disease-related liver fibrosis, the inflammatory factor TGF-β stimulates the activation of hepatic stellate cells, induces ER stress, and activates ATF6α; activated ATF6α upregulates the expression of the ER-phagy receptor FAM134B. FAM134B, through selective ER-phagy, transports TGF-β-induced misfolded collagen I from the ER to lysosomes for degradation while also regulating the intracellular transport, secretion, and fiber deposition of collagen I, inhibiting the transcription and release of pro-inflammatory and pro-fibrotic factors, such as TGF-β and TNF-α, and reducing intrahepatic inflammatory infiltration and hepatic stellate cell activation, thereby delaying hepatitis progression and blocking the occurrence and development of liver fibrosis (178). The above two pathways represent protective strategies of ER-phagy in acute and chronic liver injury, respectively. However, when ER-phagy regulatory factors are absent, the protective mechanism collapses, leading to damage. Specifically, the loss of CDK5RAP3 in the liver upregulates IL-6 and TNF-α through the NF-κB pathway and enhances NLRP3 inflammasome assembly and activation, leading to caspase-1 cleavage of pro-IL-1β to mature IL-1β, and cleavage of GSDMD to induce pyroptosis; DAMPs released from pyroptosis positively feed back to activate NLRP3, forming an “inflammation–death” cycle. At the same time, CDK5RAP3 deficiency upregulates the BAX/BCL2 ratio to activate apoptosis, and pyroptosis and apoptosis synergistically drive liver inflammation and injury (179, 180).

The pathological core of acute pancreatitis is necrosis of pancreatic acinar cells and abnormal inflammatory reactions (181), and ER-phagy in pancreatitis shows a dynamic evolution of “early protection, late failure”. In the early stage of acute pancreatitis, mild increases in p-PERK, p-eIF2α, and ATF6 cleavage effectively induce FAM134B- and CCPG1-mediated ER-phagy; inhibit the expression of apoptosis-related proteins, cleaved caspase-3 and Bax, and necrosis markers p-MLKL and Rip3; increase the level of the anti-apoptotic protein Bcl-2; and reduce the expression of the inflammatory factor IL-1β. As the disease progresses, due to excessive ROS production and Ca^2+^ homeostasis imbalance, ER stress continues to intensify, ER-phagy clearance function fails, ER stress further exacerbates, IL-1β release is promoted, and massive acinar cell apoptosis and necrosis are induced, pushing acute pancreatitis toward severe acute pancreatitis (182).

In intestinal diseases, inflammatory bowel disease is characterized by persistent NF-κB activation (183) and neutrophil over-infiltration (184). As a newly discovered ER-phagy receptor, the overexpression of UBAC2 in the Dextran Sodium Sulfate (DSS)-induced acute ulcerative colitis mouse model maintains ER homeostasis via ER-phagy, thereby inhibiting the activation of the UPR (XBP1s and CHOP) and NF-κB signaling pathways, downregulating the levels of inflammatory factors such as IL-6, TNF-α, and IL-1β, and reducing mouse weight loss, colon shortening, and tissue inflammatory damage; however, UBAC2 disease-associated mutants (such as R277C, F279S, and G293S) or LIR motif mutants have reduced binding ability to GABARAP and cannot effectively perform ER-phagy, leading to more severe colitis and inflammatory responses (67, 68). In addition, FOXO3 deficiency in inflammatory bowel disease leads to abnormal lipid droplet accumulation in neutrophils and decreased CCPG1 expression, resulting in ineffective clearance of the ER damaged by inflammatory stress. Persistent ER stress activates the NF-κB pathway via the IRE1α–TRAF2 complex, promoting the expression of pro-inflammatory factors such as IL-1β, IL-6, and TNF-α, while also enhancing neutrophil chemotaxis and migration, causing massive infiltration into colonic tissues and release of ROS and proteases, and disrupting the intestinal epithelial barrier, thereby driving colonic inflammation and even tumorigenesis (153).

In summary, the protective role of ER-phagy in inflammatory diseases of the digestive system is organ-specific and disease stage-dependent. Future studies should combine organ-specific knockout mice and disease staging models to analyze the spatiotemporal evolution of ER-phagy receptors in inflammatory digestive diseases and develop stage-specific therapeutic strategies targeting particular receptors.

### 6.4 Tumor-related diseases

As a key hub linking inflammation and tumors, CDK5RAP3 plays a central role in regulating tumor invasion and its microenvironment by inhibiting the NF-κB signaling pathway. In tumor cells, upregulation of CDK5RAP3 can, on the one hand, be stabilized by RCAD to avoid ubiquitination degradation, thereby directly binding to the RelA subunit of NF-κB and inhibiting its transcriptional activity and downstream pro-inflammatory factor expression (185). On the other hand, it can bind to NLBP, enhancing each other’s stability by inhibiting their mutual ubiquitination, thereby synergistically and persistently inhibiting the NF-κB pathway and its downstream effector molecules MMP-1 and MMP-9, ultimately reducing tumor cell invasiveness (186). In the tumor microenvironment, the loss of CDK5RAP3 activates NF-κB, leading to enhanced phosphorylation and nuclear translocation of the p65 subunit, thereby upregulating M2 macrophage polarization-related factors, such as IL-4 and IL-12, and promoting the polarization of tumor-associated macrophages toward the pro-tumor M2 phenotype (155). M2-type tumor-associated macrophages and the inflammatory cells that they recruit degrade the extracellular matrix by secreting factors, such as MMP-9, and promote epithelial–mesenchymal transition of tumor cells, collectively shaping a highly inflammatory, vascularized, and invasion-favorable microenvironment (187) and ultimately accelerating tumor progression from multiple levels.

Current studies have shown that FAM134B interacts with the chaperone protein BiP, and this interaction is crucial for hypoxia-stressed breast cancer cells. Because FAM134B itself lacks an ER lumenal domain and cannot directly sense the folding status of luminal proteins, the major chaperone BiP, located in the ER lumen, as a classic stress sensor, recognizes and binds misfolded proteins. Under hypoxic stress, BiP functionally couples with FAM134B and transmits the luminal misfolding signal to FAM134B, thereby initiating selective ER-phagy, helping cancer cells clear damaged ER fragments, alleviate ER stress, maintain homeostasis, and enhance proliferative capacity, providing a key adaptive mechanism for tumor survival in the hypoxic microenvironment (188). However, the precise molecular interface of BiP-FAM134B binding remains unclear, and whether it widely exists in other hypoxic solid tumors is unknown.

### 6.5 Nervous system diseases

Neuroinflammation is a common pathological basis of many central nervous system diseases. ER-phagy plays a core protective role by inhibiting the NF-κB/NLRP3 inflammatory axis, but its specific mechanisms show receptor selectivity and signaling pathway differences in different diseases.

In Alzheimer’s disease, TEX264-mediated ER-phagy reduces Aβ oligomer-induced ER stress, inhibiting the overactivation of the PERK-eIF2α-ATF4 and IRE1–XBP1–sXBP1 pathways, thereby downregulating downstream pro-apoptotic factors CHOP and GADD34. At the same time, ER-phagy inhibits the abnormal release of ROS and Ca^2+^, blocks JAK1–STAT3 signaling, reduces NF-κB p65 phosphorylation, thereby downregulating COX-2 and pro-inflammatory factors IL-1β and TNF-α, and upregulates anti-inflammatory factors IL-4 and IL-10, ultimately alleviating neuroinflammation (189). In aging-related neuroinflammation, CCPG1 is transcriptionally upregulated via IRE1–XBP1 and PERK–ATF4. When the CCPG1 function is impaired, ER stress levels rise sharply due to delayed clearance, and persistent ER stress triggers the NF-κB pathway via the same IRE1–XBP1 and PERK–ATF4 pathways, thereby upregulating pro-inflammatory cytokines such as TNF-α and IL-1β, thus avoiding autophagy dysfunction caused by excessive oxidation and breaking this vicious cycle (190). In contrast to the above mechanisms that clear ER stress, in post-stroke depression, FAM134B-mediated ER-phagy promotes the degradation of key components of the cGAS–STING pathway, blocking cGAS–STING signaling activation, thereby inhibiting NLRP3 inflammasome activation and the release of downstream inflammatory factors IL-1β and IL-18 (191). Unlike the first three mechanisms that indirectly inhibit NF-κB/NLRP3 by clearing ER stress or signaling platforms, CDK5RAP3 directly acts on the NF-κB subunit RelA via its C-terminal domain, binding to the Rel homology domain and blocking exposure of the nuclear localization signal of RelA, thereby inhibiting RelA nuclear translocation induced by TNF-α and other stimuli. At the same time, CDK5RAP3 can recruit protein phosphatases such as PP2A to promote dephosphorylation of RelA at Ser536 and other sites, further weakening its transcriptional activity. In addition, CDK5RAP3 binding to RelA may enhance the affinity of RelA for IκBα, stabilizing the cytoplasmic NF-κB–IκBα complex and preventing IκBα phosphorylation and degradation, ultimately inhibiting the transcription of NF-κB downstream inflammatory factors and exerting anti-inflammatory effects (192).

### 6.6 Circulatory system diseases

Genetic studies have suggested that the single-nucleotide polymorphism rs6780676 near the SEC62 gene may affect SEC62 expression or function by regulating transcription factors such as CEBPG, Foxd3, and HNF6, thereby altering ER stress-induced inflammatory responses and coronary plaque vulnerability, but genetic intervention and causal validation experiments have not yet been performed (193). To more directly verify the role of SEC62-mediated ER-phagy in inflammatory responses, in AT-1 systemic overexpression mice, impaired SEC62-mediated ER-phagy induced systemic inflammation, characterized by significantly increased plasma inflammatory factors IgA, IL-7, IL-18, and VCAM-1; extensive immunoglobulin G infiltration in peripheral tissues; and significantly increased proportions of B cells and neutrophils in peripheral blood (194). This is consistent with the known function of SEC62: SEC62 binds LC3 via its LIR motif and closes the Sec61 channel depending on high cytoplasmic Ca^2+^ signals, thereby limiting the transmission of ER stress to the NF-κB pathway and thus inhibiting the transcription of pro-inflammatory factors (115, 116).

### 6.7 Musculoskeletal system diseases

In cancer cachexia, muscular dystrophies, and muscle aging, ER stress is a common pathological hub, but its downstream signaling branches and coupling with inflammation differ by disease (195). In cancer cachexia, tumor-derived stress signals activate the PERK, IRE1, and ATF6 pathways in muscle cells. The E3 ubiquitin ligase TRAF6 indirectly enhances ER stress and induces autophagy via the NF-κB and MAPK signaling pathways, and the serine/threonine kinase PKCθ mediates autophagy-induced muscle fiber atrophy (196). The activated UPR can also directly promote the production of inflammatory factors: for example, IRE1α activates the JNK/p38 MAPK pathway via TRAF2-ASK1, PDIA4 regulates IL-6 and TNF-α, and XBP1 mediates muscle atrophy via the TLR/MyD88 axis (196). However, the UPR activation pattern differs among cachexia models; for instance, the Yoshida hepatoma model shows upregulated ATF4/GADD34 but reduced XBP1 splicing, accompanied by increased local IL-1β mRNA, indicating that the PERK–ATF4 pathway may directly upregulate the pro-inflammatory factor IL-1β independently of XBP1 (197).

The positive feedback regulation between ER stress and inflammation is a common pathological mechanism in various muscular dystrophies. Both Duchenne muscular dystrophy (DMD) and congenital myotonic dystrophy type 1 (CDM) involve direct cross-talk between the three UPR sensor branches and inflammatory signals. DMD is initiated by dystrophin deficiency, leading to cytoplasmic Ca^2+^ overload and SERCA dysfunction-induced calcium homeostasis imbalance, activating PERK/IRE1α, further activating the NF-κB-dependent classic pro-inflammatory signaling pathway, and upregulating TNF-α, IL-1β, and IL-6, resulting in chronic inflammation and fibrosis (198, 199). CDM involves two intertwined pathways: the IFN pathway, in which CUG repeat RNA activates TLR3-IRF7, triggering IFN responses, and the UPR pathway, in which CUG repeat RNA upregulates IRF7 via PERK–eIF2α–ATF4 and IRE1 amplifies inflammatory signals via NF-κB, both together inhibiting myogenesis (200). Unlike the above two subtypes, limb-girdle muscular dystrophy type 2I (LGMD2I) primarily shows upregulation of the ER chaperones GRP78 and CHOP and drives a non-large immune cell-dependent inflammatory response through MHC-I overexpression (201).

In muscle aging, ER stress interacts with inflammation through canonical and non-canonical pathways. Age-related Renin- Angiotensin- Aldosterone System (RAAS) overactivation, particularly angiotensin II, continuously activates the canonical GRP78–eIF2α–ATF4–CHOP axis, leading to abnormal ER–mitochondrial Ca^2+^/ROS exchange and upregulation of senescence-associated secretory phenotype factors such as p16INK4a, exacerbating age-related muscle loss (202). In contrast, non-canonical ER stress driven by redox imbalance, characterized by no significant upregulation of GRP78 but increased NOX2-mediated oxidative stress without a corresponding increase in catalase, mildly activates the UPR and promotes local IL-1β and IL-6 release (203). On this basis, superimposed mitochondrial dysfunction caused by OPA1 deficiency upregulates FGF21 via PERK–ATF4; FGF21, on the one hand, induces growth hormone resistance and protein catabolism and, on the other hand, activates NF-κB to elevate serum inflammatory factors such as IL-6, IL-1α, IL-1β, and TNF-α, accelerating muscle loss (204, 205). In addition, in aging combined with obesity, abnormal activation of IL-6–STAT3 signaling increases STAT3 phosphorylation at Tyr705, which in turn upregulates PERK, eIF2α, and ATF4, promoting the accumulation of the misfolded protein A11 oligomer and further activating cytochrome *c* release and caspase-9/3 activation, accelerating muscle protein degradation (206).

Although direct studies of ER-phagy in muscle-related inflammation are still few, evidence suggests that traditional Chinese massage (Tuina) upregulates FAM134B-mediated ER-phagy to clear damaged ER fragments and alleviate ER stress, thereby inhibiting muscle inflammation in skeletal muscle contusion and protecting muscle function (207). Its protective mechanism may involve ER-phagy degrading stressed ER, thereby blocking the formation of the IRE1α–TRAF2 complex downstream of ER stress, subsequently inhibiting the JNK/IKK–NF-κB signaling axis and reducing the transcriptional expression of pro-inflammatory factors such as TNF-α. Based on the above mechanisms, future studies urgently need to clarify the coupling of ER-phagy with inflammatory signals in different muscle diseases and to develop tissue-specific intervention strategies targeting ER-phagy receptors to verify whether activating ER-phagy can break the ER stress-inflammation positive feedback loop, thereby providing new strategies for treating age-related muscle atrophy, cancer cachexia, and muscular dystrophies.

### 6.8 Reproductive system diseases

**Figure 3:**
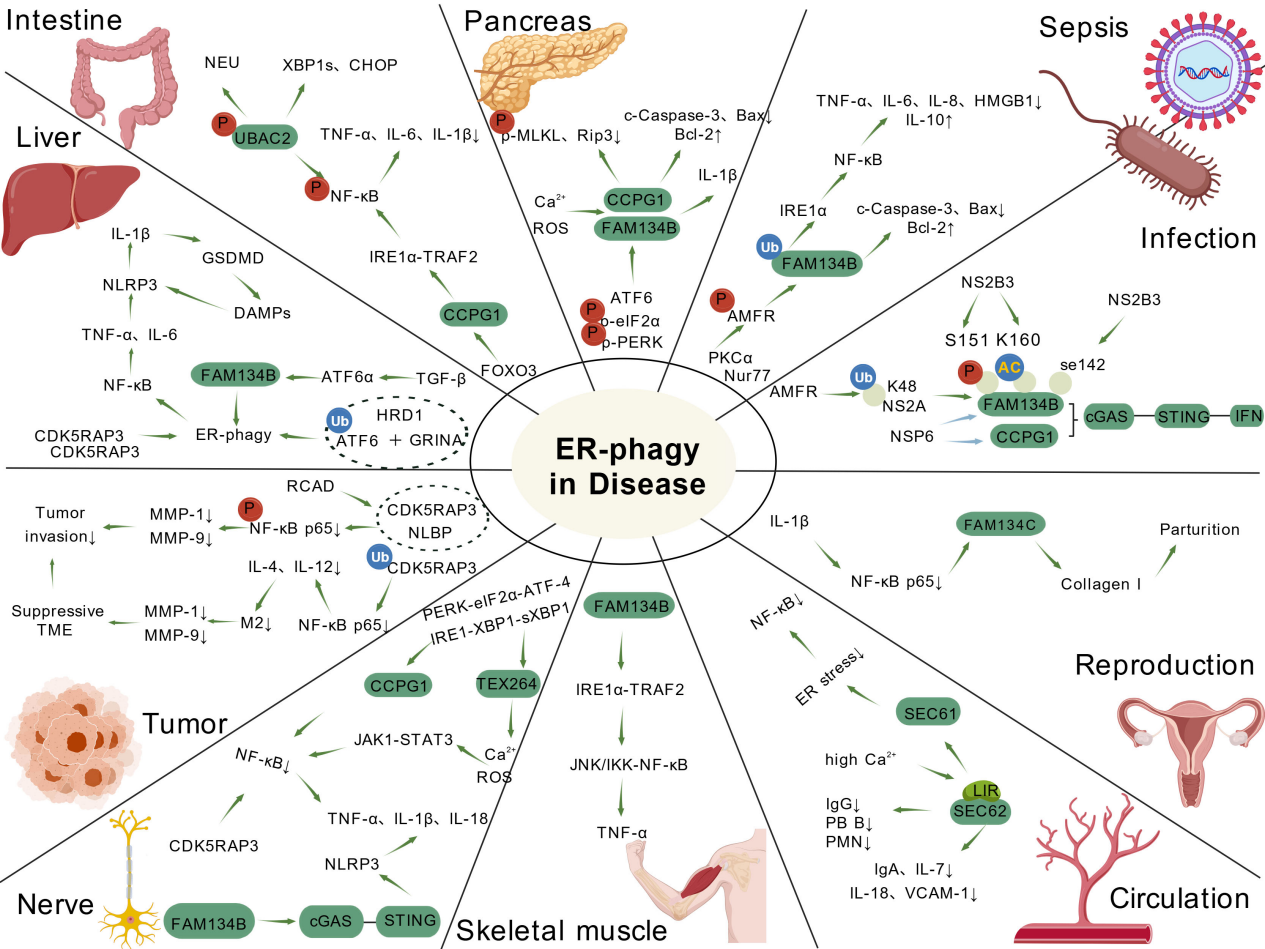
ER-phagy is closely associated with a wide range of inflammatory diseases, including infection, sepsis, digestive system diseases, tumor-related diseases, nervous system diseases, circulatory system diseases, musculoskeletal system diseases, and reproductive system diseases. ER, endoplasmic reticulum. This image was drawn by BioGDP.com.

As a major component of the human fetal membrane, the abundant collagen in the extracellular matrix of the amniotic layer gradually degrades with increasing gestational age, reaching its lowest level at term, thus facilitating membrane rupture during delivery (208). IL-1β, a key central inflammatory mediator inducing labor, binds to the IL-1R receptor on amniotic fibroblasts, on the one hand promoting the initiation of autophagy flux and, on the other hand, specifically upregulating the expression of the ER-phagy receptor FAM134C. Enhanced FAM134C-mediated ER-phagy leads to recognition, encapsulation, and transport of collagen I protein within the ER to lysosomes for degradation, resulting in decreased collagen I levels, thereby promoting membrane rupture (209). Furthermore, this study confirmed that elevated IL-1β in human amnion activates the p65 subunit of NF-κB, which then initiates the ER-phagy pathway, leading to type I collagen degradation. It is known that activated p65 may directly bind to potential κB response elements on target genes to initiate their transcription (210), but no direct evidence currently shows that FAM134C is a direct transcriptional target of NF-κB; this needs further molecular verification (see **Figure 3**).

## 7 Summary and outlook

This review systematically summarizes the molecular characteristics, regulatory mechanisms, and roles in inflammation-related diseases of mammalian ER-phagy receptors. Existing evidence indicates that ER-phagy has transcended the scope of classic organelle quality control and evolved into a key regulatory hub integrating metabolic stress, ion homeostasis, and immune responses. Unlike previous reviews that focused on a single inflammatory disease or discussed the passive clearance function of ER-phagy in isolation, this review, based on the unique pathophysiological connection between ER-phagy and inflammation, explains the multidimensional role of ER-phagy in systemic inflammatory diseases from an integrated perspective of dynamics, multiple targets, and active regulation.

ER-phagy receptors are numerous and structurally diverse, but they share several common features in inflammation regulation. Most receptors contain LIR or GABARAP interaction motif (GIM), which form the structural basis for their interaction with LC3/GABARAP. As a FAM134 family protein, FAM134A shares LIR and RHD with FAM134B/C and can functionally compensate for the loss of both under certain conditions, but its role in inflammation has not been studied; therefore, we infer that FAM134A may also participate in inflammation regulation. Future studies urgently need to use FAM134A knockout or overexpression models to fill this gap. In addition, receptor activity is commonly regulated by post-translational modifications such as phosphorylation and acetylation under ER stress or nutrient deprivation, enabling stress adaptation, active clearance of damaged ER fragments, alleviation of ER stress, and thus inhibition of overactivation of core inflammatory pathways such as NF-κB and NLRP3. Notably, CK2-mediated phosphorylation regulates receptor function in a site-dependent manner; the nature of the phosphorylation site and its position relative to the LIR domain determine whether the outcome is “activation” or “de-inhibition”. This suggests that future studies should use site-specific phosphomimetic mutations to precisely achieve selective regulation of ER-phagy, thereby exerting specific effects on inflammation.

However, different ER-phagy receptors show significant differences in inflammation regulation. At the subcellular localization level, FAM134B is mainly located in ER sheet-like structures, while FAM134C and RTN3L are enriched in tubules and three-way junctions; this localization difference suggests that they may be responsible for clearing ER fragments from different regions, thereby finely regulating inflammatory signals in space. At the substrate selectivity level, CCPG1 specifically recognizes different luminal substrates via its CIR domains, while FAM134B-2 selectively degrades ER-retained secretory proteins, indicating that different receptors have a fine division of labor in substrate recognition, thus achieving differential clearance of different inflammatory triggers. Furthermore, within the FAM134 family, FAM134A and FAM134C can regulate the collagen targeted by FAM134B, and FAM134A can compensate for the loss of FAM134B and FAM134C, but FAM134C and FAM134B cannot compensate for each other’s loss, indicating a clear difference in substrate recognition breadth among the same family, which may affect compensatory reserve capacity in different inflammatory microenvironments. At the upstream signaling regulation level, members of the same receptor family are regulated by different signals under different conditions. For example, FAM134B responds to ER stress, while FAM134C mainly responds to nutrient deprivation; even different isoforms of the same protein are regulated by different pathways under different conditions, such as FAM134B-2 being regulated by different pathways under basal and starved conditions. This regulatory heterogeneity allows cells to selectively initiate appropriate ER-phagy programs according to the type of stress, thereby controlling inflammation progression.

Although the receptor spectrum continues to expand and research depth continues to increase, the spatiotemporal regulatory mechanisms of ER-phagy receptors in different stages of the same inflammatory disease and in different disease contexts remain to be elucidated. For example, ER-phagy in acute pancreatitis shows a dynamic evolution of “early protection, late failure”. FAM134B is upregulated in infection, sepsis, and neuroinflammation disease models but is abnormally activated or cleaved and inactivated in some viral infections. However, whether the upstream regulatory signals in different tissues or pathological models are transcriptional differences (such as activation of ATF6 and XBP1s) or differences in post-translational modification sites (such as phosphorylation and ubiquitination) still lacks systematic research. Therefore, how to analyze the dynamic evolution of ER-phagy receptors during inflammatory progression, achieve precise monitoring of ER-phagy intensity, and develop small-molecule compounds targeting the LIR domains or phosphorylation modification sites of specific receptors, while avoiding interference with systemic immune homeostasis and while enhancing clearance efficiency of damaged ER, is precisely the core challenge for achieving clinical translation and precision anti-inflammatory intervention.
